# Supplementary material for: Assessing the Clinical Impact of the SARS-CoV-2 Gamma Variant on Intensive Care Unit Admissions: Insights from a Reference Hospital in Northeastern Brazil
Source: Viruses. 2024 Mar 20;16(3):467. doi: 10.3390/v16030467 (PMC10974300; doi:10.3390/v16030467)
Supplement: Supplementary file 1 [file viruses-16-00467-s001.zip › Tables S1 & S2.pdf]

Supplementary Table S1. Reference ranges for laboratory indicators utilized by São Rafael Hospital's laboratory.

Parameters	Reference value
CRP (mg/L)	< 10,0 mg/L
D-dimer (ng/mL)	< 500 ng/mL
Fibrinogen (mg/dL)	200 a 400 mg/dL
APTT (seg)	25.0 to 40.0 seconds
NtProBNP, pg/mL	Age <50: 450 pg/mL<br>Age 50-75: 900 pg/mL<br>Age > 75 yars: 1800 pg/mL
Troponin 1, ng/mL	<0.034 ng/mL
LDH, U/L	120 a 246 U/L
Ferritin, ng/mL	17.9-464.0 ng/mL
Bilirubin, mg/dL	0,2 to 1,3 mg/dL
ALP,U/L	38 to 126 U/L
CK, U/L	Female: 30 a 135 U/L<br>Male: 55 a 170 U/L
GGT, U/L	Female: 12 to 43 U/L<br>Male: 15 to 73 U/L
ALT, U/L	Female: < 35 U/L<br>Male: < 50 U/L
AST, U/L	17 to 59 U/L
Creatinine, mg/dL	0.70 a 1.20 mg/dL
Urea, mg/dL	19 a 43 mg/dL

Abbreviations: CPR: C-Reactive Protein; APTT: Activated Partial Thromboplastin Time; ALP: Alkaline Phosphate; CK: Creatine Kinase; GGT: Gamma-glutamyl Transferase; ALT: Alanine Aminotransferase ; AST: Aspartate aminotransferase

Supplementary Table S2. Optimized primer list for the SARS-CoV-2 variant detection using the PCR method for genotyping

ID	DNA sequences (5'-3')
**F3-69/70del forward**	ACGTGGTGTTTATTACCCTGACA
**R3-69/70del reverse**	TGGTAGGACAGGGTTATCAAAC
**S3-69/70del Probe**	Cy5-CCATGCTATACATGTCTCTGGGA-BHQ2
**F3-Orf1a-del forward**	TGCCTGCTAGTTGGGTGATG
**R3-Orf1a-del reverse**	AGTAACACTACAGCTGATGCA
**S3-Orf1a-del-Probe**	HEX-TAGTTTGTCTGGTTTTAAGCTAAAAGACT-BHQ1
**N1-F forward**	GACCCCAAAATCAGCGAAAT
**N1-R reverse**	TCTGGTTACTGCCAGTTGAATCTG
**N1- Probe**	FAM-ACCCCGCATTACGTTTGGTGGACC-BHQ1
