# Supplementary figures and images for: Assessing the Clinical Impact of the SARS-CoV-2 Gamma Variant on Intensive Care Unit Admissions: Insights from a Reference Hospital in Northeastern Brazil
Source: Viruses. 2024 Mar 20;16(3):467. doi: 10.3390/v16030467 (PMC10974300; doi:10.3390/v16030467)

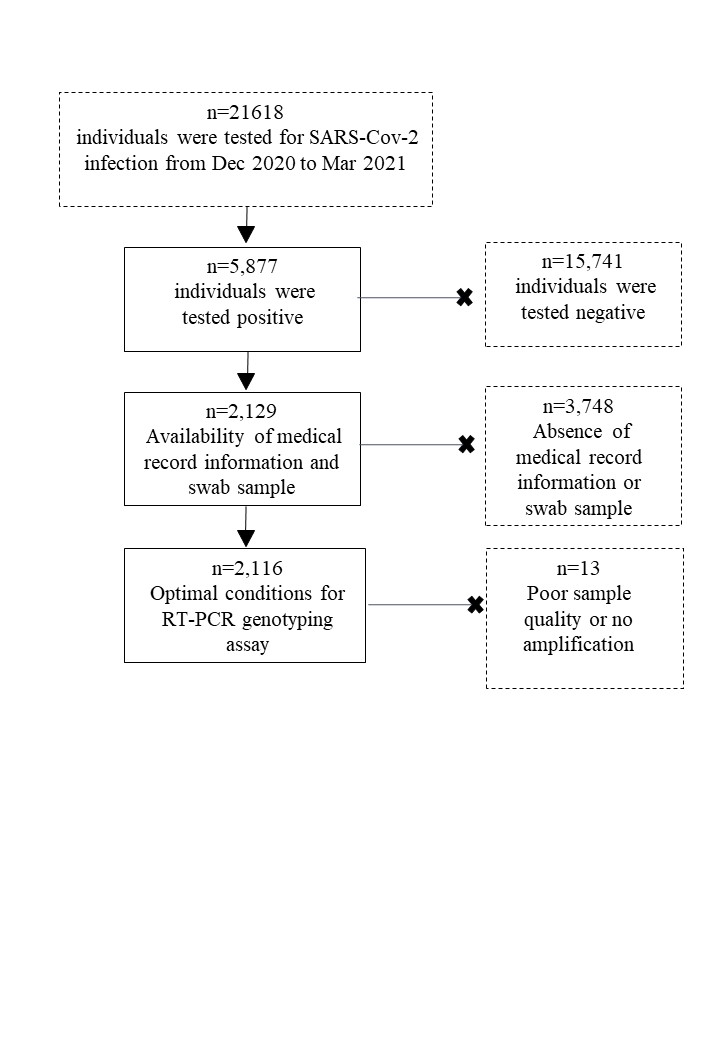

Supplement: Supplementary file 1 [file viruses-16-00467-s001.zip › Figure S1.jpg]
